# Sperm retrieval and concomitant tumor resection in azoospermic men with congenital adrenal hyperplasia and bilateral testicular adrenal rest tumors: a case report

**DOI:** 10.1007/s10815-016-0665-9

**Published:** 2016-02-09

**Authors:** Parviz K. Kavoussi, Roxanne B. Summers-Colquitt, Kate C. Odenwald, Megan Kressin, Keikhosrow M. Kavoussi, Thomas B. Pool, Shahryar K. Kavoussi

**Affiliations:** Austin Fertility & Reproductive Medicine/Westlake IVF, 300 Beardsley Lane, Building B, Suite 200, Austin, TX 78746 USA; Department of Pathology, St. David’s South Austin Medical Center, 901 W. Ben White Blvd, Austin, TX 78704 USA

**Keywords:** Congenital adrenal hyperplasia, Testicular adrenal tumor, Sperm retrieval

## Abstract

**Purpose:**

The objective of this study was to offer a new treatment approach for sperm retrieval simultaneously with tumor resection in azoospermic men with congenital adrenal hyperplasia (CAH), orchialgia, and bilateral testicular adrenal rest tumors (TARTs) who fail to respond to medical treatment.

**Methods:**

This is a retrospective chart review from a couple’s fertility center.

**Results:**

Between May 2013 and May 2015, two azoospermic men with CAH and bilateral TARTs, with orchialgia, and desire to conceive underwent bilateral TART resection in the same surgical setting as sperm retrieval after remaining azoospermic with normalization of gonadotropins with treatment with human chorionic gonadotropin (hCG). Both men had adequate sperm retrieved for in vitro fertilization/intracytoplasmic sperm retrieval (IVF/ICSI) at the time of bilateral TART resections. They had complete TART resections with resolution of orchialgia. The wife of one patient had a successful pregnancy with use of retrieved sperm resulting in a live birth, and the sperm from the other man is cryopreserved for future use.

**Conclusions:**

It is feasible to perform successful sperm retrieval simultaneously with TART resection in azoospermic men with CAH after medical treatments with persistent azoospermia, rather than subjecting these men to multiple invasive procedures.

## Introduction

Men with congenital adrenal hyperplasia (CAH) have impaired cortisol and aldosterone production, resulting in increased adrenocorticotropic hormone (ACTH) production and an associated hyperplasia of the adrenal glands with overproduction of adrenal androgens [1, 2]. Some men with CAH will develop benign testicular adrenal rest tumors (TARTs) [3, 4]. TARTs are typically bilateral and originate in the rete testis [5–7]. TARTs commonly result in obstructive azoospermia, destroy normal testicular parenchyma with growth, and may cause orchialgia. There are reports of sperm retrieval via testicular aspiration, with TARTs in situ, to use for in vitro fertilization/intracytoplasmic sperm injection (IVF/ICSI) [8]. There are also reports of men remaining azoospermic after TART resections [9]. The objective of this study was to evaluate the effectiveness of simultaneous sperm retrieval and TART resections in cases of persistent azoospermia following medical treatment, with the goal of offering a new treatment approach which minimizes the number of procedures performed in such patients, in order to secure sperm to assist with fertility as well as to resect TARTs to relieve orchialgia and prevent further tissue destruction.

## Materials and methods

Sperm retrieval was performed concomitantly with bilateral TART resection in two men with CAH, orchialgia, and azoospermia between May 2013 and May 2015 by a single surgeon (PKK). Procedures were performed through bilateral inguinal incisions with vascular control of the spermatic cords in an oncologically sound manner. Testicular tumors were resected en-bloc after bivalving the testicles in a vascular preserving manner. Once frozen section pathology revealed no evidence of malignancy, sperm retrieval was performed and confirmed by two embryologists in the operating room and the testicles were closed, placed back in anatomic positions, and the incisions were closed in layers. The outcomes measured included successful sperm retrieval deemed adequate for IVF/ICSI, complete en-bloc resection of TARTs, and post-operative resolution of orchialgia. A retrospective chart review was performed to evaluate outcomes.

## Results

**Fig. 1:**
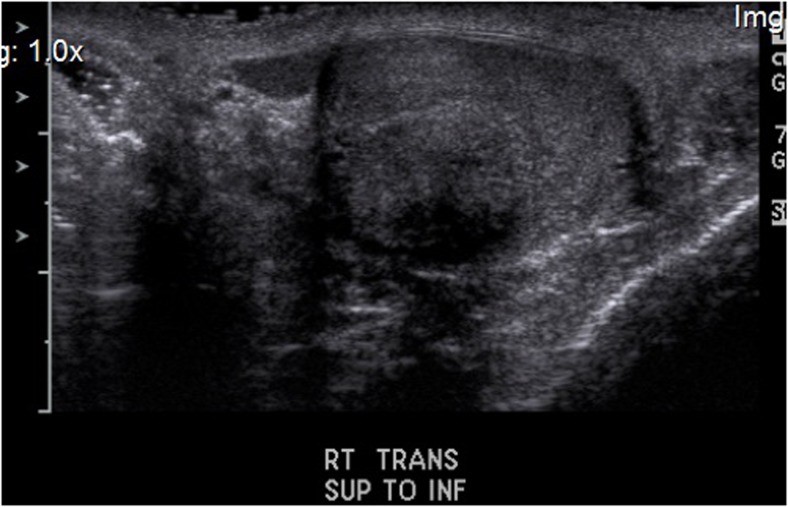
Sonographic appearance of TART

**Fig. 2:**
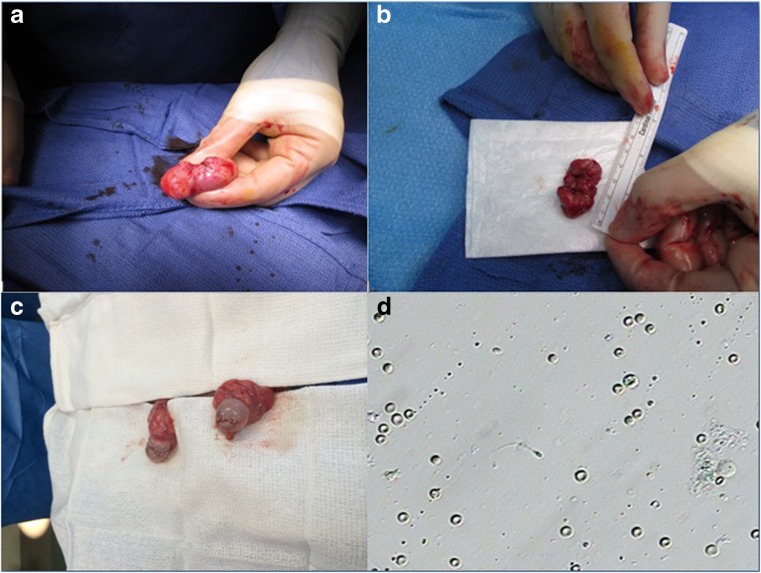
**a** TART in situ. **b** En-bloc resected specimen of TART. **c** Bilateral testicles after reconstruction and repair following TART resections. **d** Microscopic appearance of retrieved sperm at the time of surgery

**Fig. 3:**
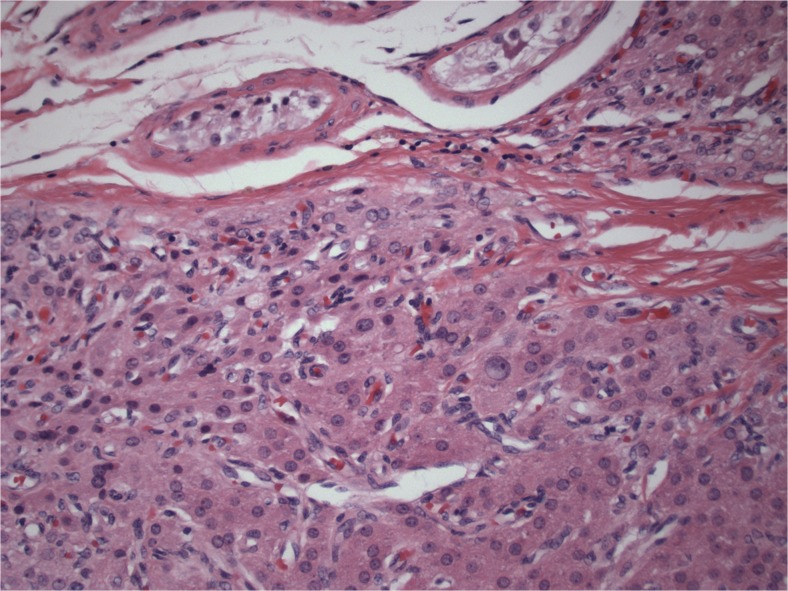
Pathologic architecture consistent with adrenal rest tumor

Two men, ages 35 and 40, with previous diagnoses of classic CAH with 21-hydroxylase deficiency who were under long-term care of endocrinologists were referred for evaluation of orchialgia and testicular masses. They were each found to have bilateral TARTs as well as azoospermia on two semen analyses. They underwent scrotal ultrasounds which revealed bilateral testicular tumors at the level of the rete testes (Fig. 1). They continued treatment with dexamethasone and were found to be hypogonadotropic with undetectable serum follicle stimulating hormone (FSH) and luteinizing hormone (LH) levels. They were treated with subcutaneous injections of human chorionic gonadotropin (hCG) 2000 IU twice a week and on repeat testing 1 month later, were found to have normalized FSH and LH levels (FSH 6.3, 5.8 and LH 1.5, 2.3). Repeat semen analyses 3 months after normalization of gonadotropins revealed persistent azoospermia, indicating an obstructive process consistent with the location of TARTs at the rete testes. They underwent bilateral en-bloc TART resections with simultaneous successful sperm retrieval. Once frozen section pathology revealed no evidence of malignancy, the testicles were closed and preserved. Sperm was cryopreserved in both cases (Fig. 2). Permanent section pathology revealed architecture consistent with adrenal rests with negative margins (Fig. 3).

One man proceeded with use of his sperm for IVF/ICSI, and his wife achieved a successful pregnancy resulting in a live birth. The other man had his sperm cryopreserved for future use. Both men had complete resolution of orchialgia after recovery.

## Discussion

CAH is the most commonly found genetic steroidogenic disorder affecting fertility. Approximately 1 in 16,000 individuals has classic CAH, which makes it one of the most common autosomal recessive human genetic diseases [1]. This inherited disorder impacts the steroid synthesis of the adrenal gland. In greater than 90 % of CAH cases, the cause is CYP21 (21-hydroxylase) deficiency [5, 10, 11]. There are a number of pathological mechanisms which may contribute to subfertility in men with CAH. Hypogonadotropic hypogonadism occurs due to high adrenal androgen concentrations. These markedly elevated adrenal androgens may also aromatize to estrogens peripherally, resulting in further suppression of gonadotropin secretion and, in turn, impaired function of Leydig cells [2]. A decrease in the number of germ cells in these patients has been reported [12]. Glucocorticoid treatment alone can be effective in restoring normal spermatogenesis [13, 14]; however, men with TARTs that cause obstruction at the level of the rete testis are at high risk of persistent azoospermia.

TARTs are identified in 27 to 47 % of men with classic CAH [3, 4]. TARTs may develop in childhood in boys with CAH. Twenty-one percent of boys, between the ages of 2 and 10 years, with classic CAH were found to have TARTs at screening [15]. These tumors are characterized by hypertrophic adrenocortical remnants in focal areas. They result in response to prolonged stimulation by ACTH. TARTs destroy normal testicular parenchyma and cause obstruction [5]. Treatment with dexamethasone has been reported to decrease the size of TARTs in some men and even have resulted in complete resolution in some cases [16]; however, glucocorticoid treatment with suppression of ACTH secretion is not always successful in reducing tumor sizes, even with intensifying doses [6, 17, 18]. It has even been suggested that glucocorticoid overtreatment may have an adverse effect on spermatogenesis by suppression of the hypothalamic-pituitary-gonadal axis, and there may be individual variation in responses [19]. The end stage, irreversible effect of a TART is tubular hyalinization with obstruction of the lumen and complete loss of germ cells and Sertoli cells [5]. Previously, testicular biopsy was suggested prior to surgical treatment; however, such a biopsy provides information about a very limited area of the testis and subjects the patient to an additional procedure [5]. Testis sparing surgery has been described in men with TARTs of significant size [17, 20]. There has been a reported case of conversion from azoospermia to severe oligospermia in a man with a solitary testis and small TART which was apparently not completely obstructive, who was treated with hCG and FSH [21]. It has been reported that testicular sparing surgery for TARTs resulted in persistent azoospermia post-operatively, presumably due to further obstruction of the rete testis or from prior destruction of the rete testis from the TARTs. The patient in that report had a testicular biopsy with sperm retrieval indicating spermatogenesis and was successful with IVF/ICSI following TART resection [9]. Although a report of testicular aspiration for sperm retrieval for use with IVF/ICSI with TARTs in situ has been established, the tissue-destroying TARTs, which are not always amenable to medical therapy, were not resected in that report [8]. A single patient case report has also been published revealing treatment of an azoospermic man with bilateral TARTs who remained azoospermic following 6 months of glucocorticoid treatment who was then treated with mitotane to induce chemical adrenalectomy. Mitotane treatment ultimately resulted in TART shrinkage and an increased number of sperm present in the ejaculate after 8 months of treatment. After 2 years of mitotane treatment, IVF/ICSI resulted in a pregnancy [22]. Although this treatment regimen has the potential of avoiding surgery, mitotane side effects included hyponatremia and weight loss. This may be a feasible approach for some couples, but if the female partner’s evaluation reveals diminished ovarian reserve or her fertility potential is in decline due to age, the timeframe of response to this treatment may offer limited use for the goal of conception in such couples. Family planning and the number of children the couple desires to have may play a role with this timeframe of treatment.

Our proposed treatment approach in men with CAH, bilateral TARTs, azoospermia, and orchialgia is to begin with medical treatment with glucocorticoids. Serum gonadotropins should be evaluated. If hypogonadotropic, the man should be treated with hCG, and FSH when indicated depending on gonadotropin response. When gonadotropins and testosterone normalize, a repeat semen analysis should be performed no sooner than 3 months following normalization of the hormonal milieu. When azoospermia persists, mitotane treatment may be considered depending on the female partner’s ovarian reserve status and the couple’s family planning. In cases where mitotane therapy may compromise the probability of successful pregnancy by delaying fertility treatment, the side effects of mitotane are not tolerable, or the man fails to respond, it is reasonable to perform bilateral TART resection through inguinal incisions with frozen section confirmation and simultaneous sperm retrieval in order to minimize the number of procedures the man undergoes. This accomplishes the goals of TART resection and sperm retrieval in one surgical setting. Although this sample size of two case reports is a limitation, it provides a novel approach for treatment of these men where there is a paucity of data and limited options described in the literature.

## Conclusions

To our knowledge, these are the first cases reported of azoospermic men with CAH, orchialgia, and bilateral TARTs who underwent successful sperm retrievals concurrently with bilateral TART resections. This treatment approach allows for sperm retrieval and TART resection in one surgical setting which would seem to be the optimal approach in certain clinical scenarios, rather than subjecting such patients to multiple separate procedures.
